# The microRNA-Processing Enzyme Dicer Is Essential for Thyroid Function

**DOI:** 10.1371/journal.pone.0027648

**Published:** 2011-11-21

**Authors:** Daniela Frezzetti, Carla Reale, Gaetano Calì, Lucio Nitsch, Henrik Fagman, Ola Nilsson, Marzia Scarfò, Gabriella De Vita, Roberto Di Lauro

**Affiliations:** 1 IRGS Biogem s.c.ar.l., Ariano Irpino, Italy; 2 Dipartimento di Biologia e Patologia Cellulare e Molecolare, Università degli Studi di Napoli ‘Federico II’, Naples, Italy; 3 Istituto di Endocrinologia ed Oncologia Sperimentale CNR, Naples, Italy; 4 Sahlgrenska Cancer Center and Department of Pathology, Sahlgrenska Academy at the University of Gothenburg, Gothenburg, Sweden; French National Center for Scientific Research - Institut de Biologie Moléculaire et Cellulaire, France

## Abstract

Dicer is a type III ribonuclease required for the biogenesis of microRNAs (miRNAs), a class of small non-coding RNAs regulating gene expression at the post-transcriptional level. To explore the functional role of miRNAs in thyroid gland function, we generated a thyrocyte-specific Dicer conditional knockout mouse. Here we show that development and early differentiation of the thyroid gland are not affected by the absence of Dicer, while severe hypothyroidism gradually develops after birth, leading to reduced body weight and shortened life span. Histological and molecular characterization of knockout mice reveals a dramatic loss of the thyroid gland follicular architecture associated with functional aberrations and down-regulation of several differentiation markers. The data presented in this study show for the first time that an intact miRNAs processing machinery is essential for thyroid physiology, suggesting that deregulation of specific miRNAs could be also involved in human thyroid dysfunctions.

## Introduction

MicroRNAs (miRNAs) are the main players of a recently discovered layer of gene regulation in eukaryotic organisms. These are short (19–22 nucleotide-long) non-protein-coding RNAs that negatively regulate gene expression at the post-transcriptional level by binding to complementary sequences in the 3′-untranslated regions (UTRs) of target mRNAs. As a consequence, they inhibit protein synthesis by preventing translation and/or promoting mRNA degradation [1]. miRNAs thus are involved in fine-tuning target gene expression, working in parallel with transcriptional regulatory processes.

miRNAs have been identified in many different organisms, from plants to humans, and it is estimated that they regulate 30% of known genes [2]. Some miRNA species are ubiquitously expressed whereas other display tissue-specific expression pattern. miRNA expression profiles are highly dynamic during embryonic development and in adulthood. Misexpression of miRNA can perturb embryogenesis, organogenesis and tissue homeostasis [3].

miRNAs are usually transcribed as primary miRNA (pri-miRNA) that are subsequently cleaved by the nuclear enzyme Drosha and its partner DGCR8 into 60–70 nucleotide long precursors miRNAs (pre-miRNAs) [4]. Pre-miRNAs are then transported to the cytoplasm where the cytoplasmic RNAse Dicer and its partner TRBP cleave them into a 20–25 nucleotide long mature miRNA duplex. The double-stranded miRNA then assembles into the RNA-induced silencing complex (RISC). One of the strands is degraded whereas the mature miRNA strand acts as a guide to direct RISC to target mRNAs by base pairing with sequences in their 3′ UTR [5], [6]. Based on current knowledge about miRNAs biosynthesis, it is expected that the lack of functional Dicer alleles is able to block the maturation of pre-miRNAs to its mature form, thus perturbing any regulatory circuit in which miRNAs are involved [7].

Mice lacking Dicer die at E7.5 [8], thus to elucidate the role of miRNAs in tissue development and function at later stages, Dicer conditional alleles have been generated. Dicer inactivation in limb, lung, central nervous system, prostate and muscle results in severe morphological defects and aberrant development [9]–[12]. Moreover Dicer deficiency impairs proper function of several organs, such as the heart, liver, kidney and pancreas [13]–[18]. We have recently shown that neoplastic transformation of thyroid is associated to a deregulation of several miRNAs, and that miR-21 contributes to the proliferation of thyroid cancer cells [19]–[21]. However the role of Dicer and miRNAs in thyroid gland development and function remains to be explored.

Thyroid gland development in mice begins at E8.5 in the posterior region of the pharyngeal floor in a small patch of cells marked by the simultaneous expression of a unique combination of transcription factors [22]. However, the expression of genes essential for thyroid hormones synthesis, such as Na/I symporter (Nis), thyroglobulin (Tg) and thyroperoxidase (Tpo), is detected only at around E15. Shortly after, at E16, thyroid epithelial cells organize themselves in follicles [22]. At birth, the thyroid gland is able to produce and release thyroid hormones, although the regulation of its growth and function by the hypothalamic-pituitary axis is fully active in the mouse only after birth [23].

Hormone synthesis also requires cell polarization with segregation of membrane proteins to either apical or basolateral membrane domains. Furthermore, the luminal content of the follicles needs to be isolated from the tissue interstitium. This is accomplished by firm cell-cell adhesion, that is partly mediated by adherens junction proteins as the cadherins. These are expressed in thyroid follicular cells and are important for the maintenance of follicular polarity [24].

In the present work, we investigate the *in vivo* requirement for miRNAs in thyroid development, differentiation and function by the use of a mouse model of thyroid-specific Dicer inactivation. Pax8 is one of the earlier markers of the thyroid cells during development, starting its expression at E8.5 when the specification of the thyroid bud begins. We have conditionally inactivated a floxed Dicer allele in thyroid epithelial cells during embryonic development by a Cre recombinase expression controlled by the Pax8 promoter. In knockout embryos development and/or differentiation of the thyroid gland appears unaffected. In contrast, soon after birth, Dicer knockout mice develop severe hypothyroidism, accompanied by a progressive derangement of thyroid follicular structure, thus showing that Dicer function is mandatory for normal thyroid function in later life. To the best of our knowledge, the data presented here demonstrate for the first time that a normal miRNA metabolism is essential for maintaining the normal thyroid gland structure and function in the adult.

## Results

### Thyroid-specific inactivation of Dicer

**Figure 1:**
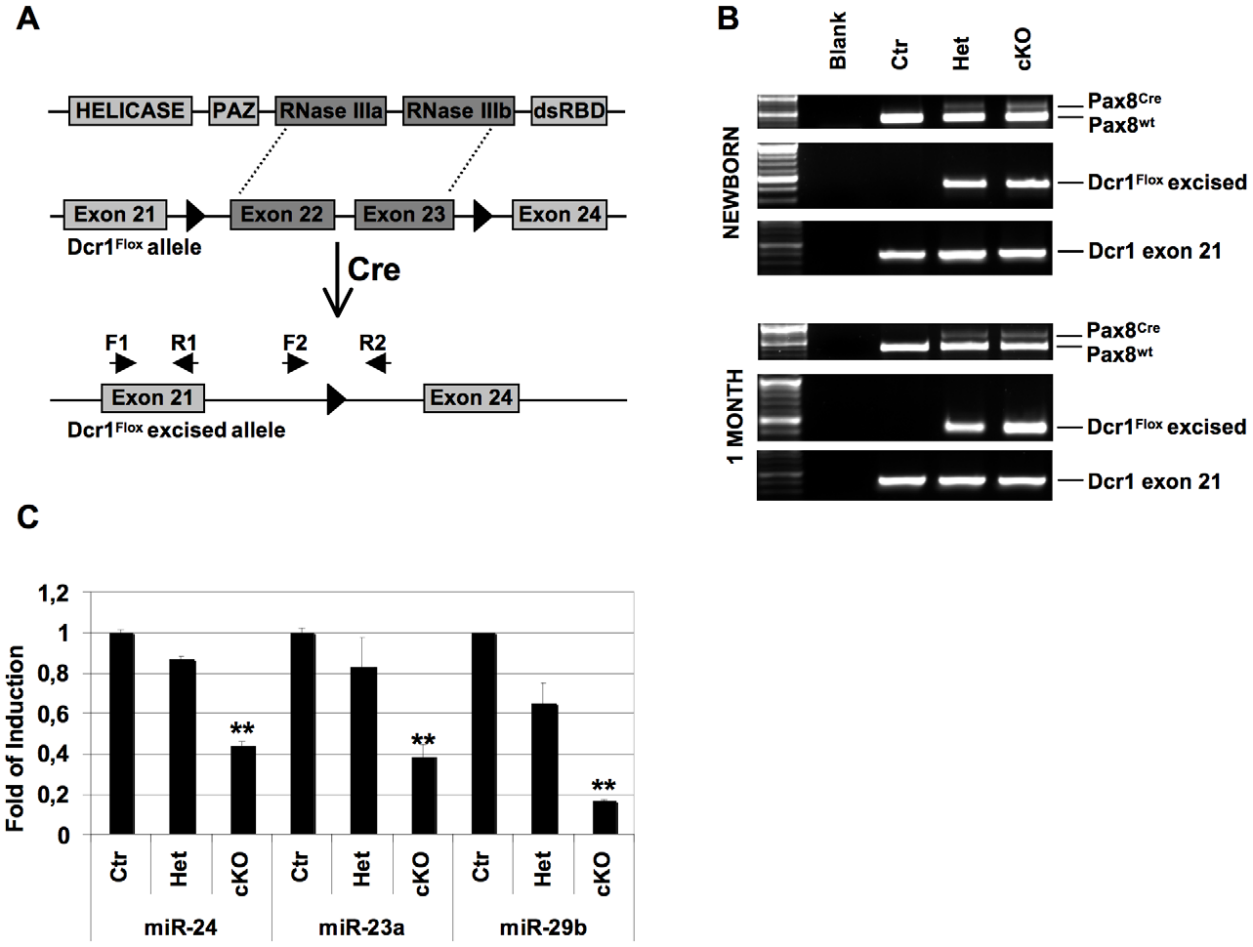
Generation of thyroid conditional Dicer knockout mice. (A) Gene targeting strategy for Dicer conditional inactivation achieved by Cre mediated removal of RNase III domains. (B) PCR analysis of thyroid genomic DNA extracted from newborn and one month old mice of the indicated genotypes. Pax8 alleles were amplified with a primer set enabling to distinguish the wild-type allele (lower band) from the Cre-encoding one (upper band). For the amplification of Dicer alleles two different primer sets were used, one (F2-R2) amplifying the floxed region (Dcr1^Flox^ excised, middle panels), and the other (F1-R1), amplifying part of the exon 21, used as a control (bottom panels). Dicer-amplifying primers positions are shown in A. (C) Q-PCR analysis of mature miR-24, miR-23a, miR-29b relative expression in mice thyroids. P-value (*:p<0,05; **:p<0,005) was calculated with Student's t-test comparing Ctr to cKO mice. Ctr denotes Pax8^Cre/+^ mice, used as controls.

In order to understand the global involvement of miRNAs in thyroid development and function, we applied the Cre-LoxP system to disrupt Dicer expression specifically in the thyroid. Mice homozygous for a floxed Dicer allele (Drc1^Flox/Flox^), which do not show phenotype abnormalities [13], were crossed to the knock-in Pax8^Cre/+^ mice, which express Cre recombinase under the control of the endogenous Pax8 gene promoter [25]. Double heterozygous Drc1^Flox/+^;Pax8^Cre/+^ (Het) progeny were identified and mated with Drc1^Flox/Flox^ mice to obtain thyroid-specific Dicer deficient animals Drc1^Flox/Flox^;Pax8^Cre/+^ (cKO). Mutant offspring were observed at the expected Mendelian frequency. Site-specific recombination of the floxed allele, which results in the removal of the majority of the catalytic RNAse IIIa and RNAse IIIb domains (fig. 1A), was observed only in heterozygous and homozygous Dicer floxed mice expressing Cre recombinase (fig. 1B). In Ctr (Dcr1^Flox/Flox^) and Het mice, the product of Dcr^Flox^ unexcised alleles are not observed presumably because its lenght (3.9 Kb) does not allow the formation of appreciable amounts of product. We analyzed by Q-PCR the expression of three miRNAs (miR-24, miR-23a and miR-29b) known to be expressed in thyroid epithelial cells [20]. As it is shown in fig. 1C all three miRNAs are expressed at significantly reduced levels in thyroids from cKO mice, compared to control mice. These data demonstrate that conditional inactivation of Dicer in thyroid results in decrease of mature miRNA expression.

### Dicer conditional knockout mice develop hypothyroidism

**Figure 2:**
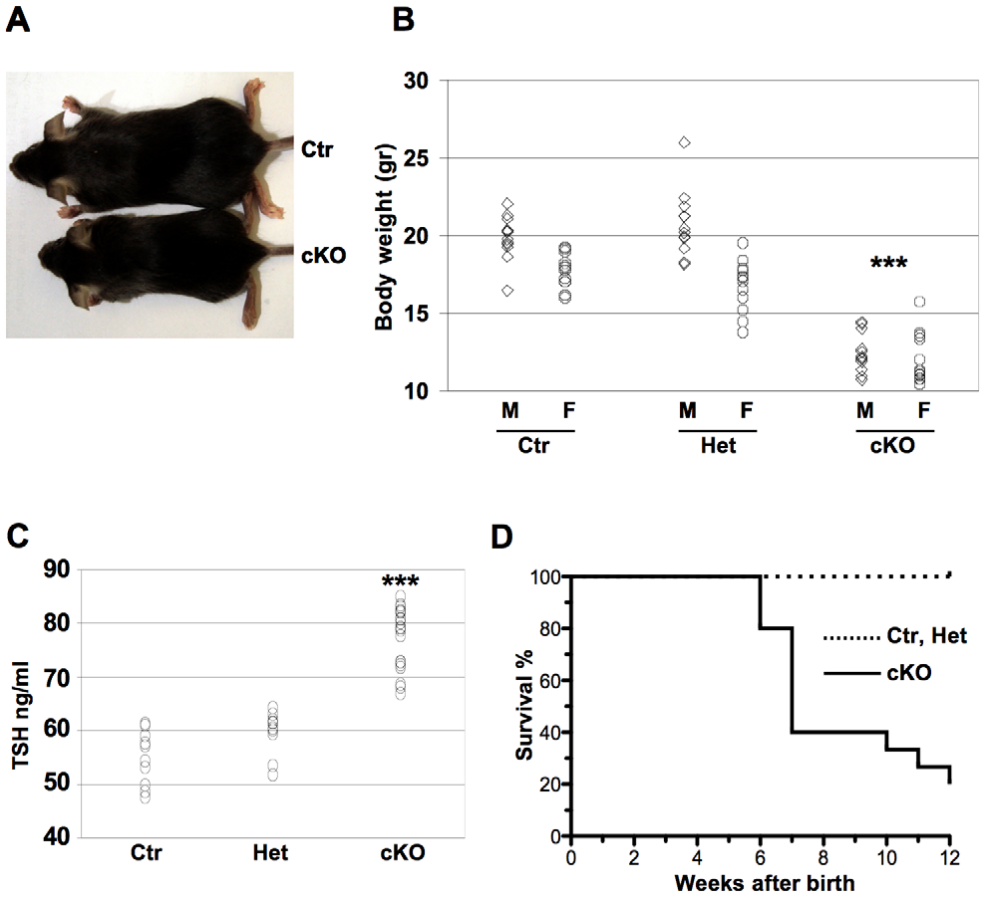
Hypothyroidism in Dicer cKO mice. (A) One month old cKO mice are smaller than Ctr littermates. (B) Body weight of male and female mice at one month after birth (n = 12 for each sex and genotype). P-value (***:p<10e-11) was calculated with Student's t-test comparing both Ctr and Het to cKO mice of the same sex. (C) ELISA assay of TSH serum level of Ctr (n = 10), Het (n = 10) and (n = 20) cKO mice at one month after birth (the same number of female and male mice have been analyzed). P-value (***:p<10e-13) was calculated with Student's t-test comparing both Ctr and Het to cKO mice. (D) Survival rates for Ctr, Het and cKO mice (n = 15 for each) during 12 weeks after birth. In all panels Ctr denotes Dcr1^Flox/Flox^ mice, used as controls.

Dicer mutant mice appeared undistinguishable from the control mice at birth (not shown). In contrast, at one month of age, they were clearly smaller than control littermates (fig. 2A) and both male and female cKO mice displayed a significantly reduced body weight compared to control and double heterozygous mice (fig. 2B). In order to investigate if the reduced body weight could be due to thyroid dysfunction, TSH levels were analyzed in peripheral blood. As compared to control Drc1^Flox/Flox^ and double heterozygous, cKO mice presented a significant increase of serum TSH indicative of hypothyroidism (fig. 2C). It is thus likely that the observed reduction of body weight is at least partly due to thyroid hormone deficiency. Moreover cKO mice had a severely shortened life span, with 80% of mice dying before 12 weeks of age (fig. 2D).

### Thyroid morphogenesis is not affected in conditional Dicer knockout embryos

**Figure 3:**
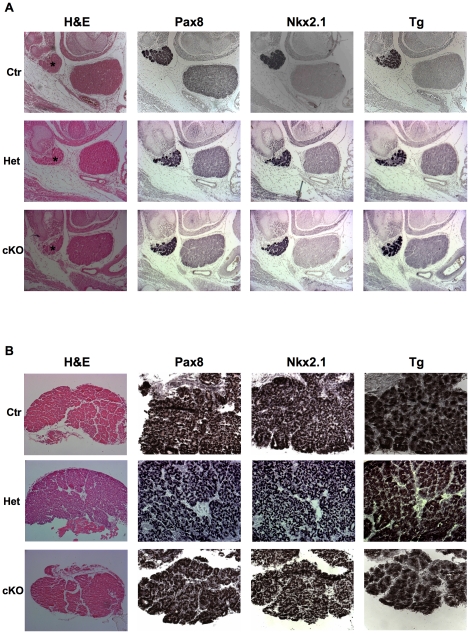
Thyroid morphology and differentiation in Dicer cKO E15.5 embroys and newborn mice. (A) Hematoxylin and eosin (H&E) staining and immunohistochemistry for Pax8, Nkx2.1 and Tg of Ctr, Het and cKO mice embryos at E15.5 (100× magnification). (B) The same analysis as in A was performed on thyroids of newborn mice (200× and 400× magnification for H&E staining and immunohistochemistry, respectively). In both panels Ctr denotes Pax8^Cre/+^ mice, used as controls.

In order to evaluate if Dicer inactivation affects thyroid embryonic development, E15.5 embryos were analyzed, as Cre recombinase in Pax8^Cre/+^ mice is active from E8.5 [25]. At E15.5 no alterations in thyroid size and morphology could be detected (fig. 3A). Moreover, immunohistochemical staining showed that Dicer mutant mice express both early (Pax8 and Nkx2.1) and late (Tg) thyroid differentiation markers, similarly to control mice (fig. 3A). We performed tha same analysis in newborn mice, showing that the expression patterns were not affected, while mild morphological alterations of follicular structure could be discerned specifically in mutant mice (fig. 3B). Taken together, these data suggest that intact Dicer activity is not required for embryonic thyroid development and differentiation.

### Dicer is essential for maintaining normal postnatal thyrocyte morphology and differentiation

**Figure 4:**
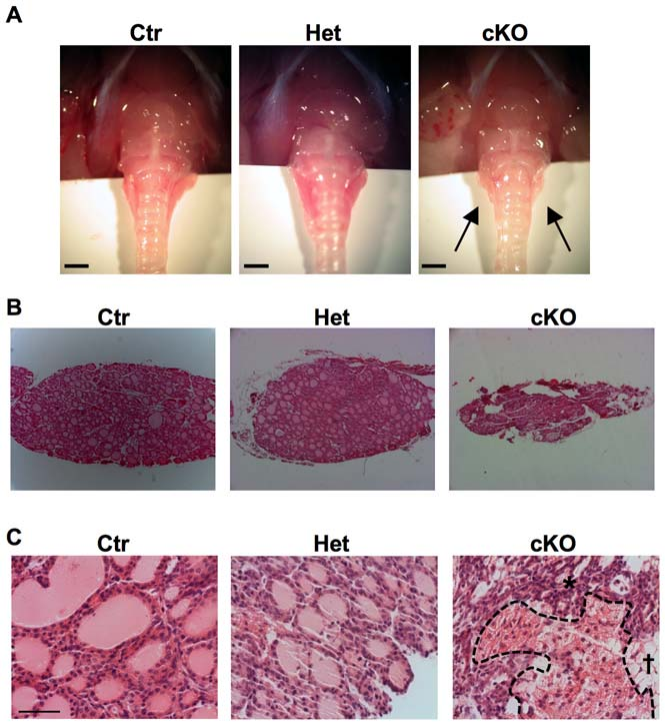
Thyroid morphology in Dicer cKO mice at one month after birth. (A) Thyroid morphology in Ctr, Het and cKO mice at 1 month (scale bar: 1 mm). The thyroid gland is evidently smaller in cKO mice (black arrows) than in controls. (B) H&E staining of Ctr, Het and cKO thyroid sections at 1 month (100× magnification). cKO thyroid glands present a severely disorganized follicular architecture. (C) H&E staining of cKO thyroid sections with oxyphilic cells. Ctr and Het sections are shown as control (scale bar: 50 µm). In the cKO section it is possible to distinguish oxyphilic (encircled area), follicular *) and vacuolated (†) cells. Ctr denotes Dcr1^Flox/Flox^ or Pax8^Cre/+^ mice, used as controls.

The effects of Dicer conditional inactivation on thyroid morphology and differentiation were analyzed in mice at one month of age. The thyroid glands of Dicer knockout mice at 4 weeks of age were considerably smaller than control and heterozygous mice (fig. 4A). Strikingly, the follicular architecture of mutant mice was almost completely destroyed (fig. 4B). As can be seen in figure 4C, thyroid cells in cKO mice are morphologically heterogenous. Only few follicular structures are present and these are of highly variable diameter with irregular outlines (figure 4B and C). Most lumina are small sized and the nuclear to cytoplasmic ratio of the surrounding cells is increased (fig. 4C). No infiltration of inflammatory cells or increased fibrosis have been noticed. In contrast to the normal gland, strongly oxyphilic cells with abundant granular cytoplasm are detected, either in clusters or scattered in the parenchyma (fig. 4C). In some cells the cytoplasm furthermore appears to be highly vacuolated (fig. 4C). Even though the gland size is clearly reduced, no increased rate of apoptosis could be detected (data not shown).

**Figure 5:**
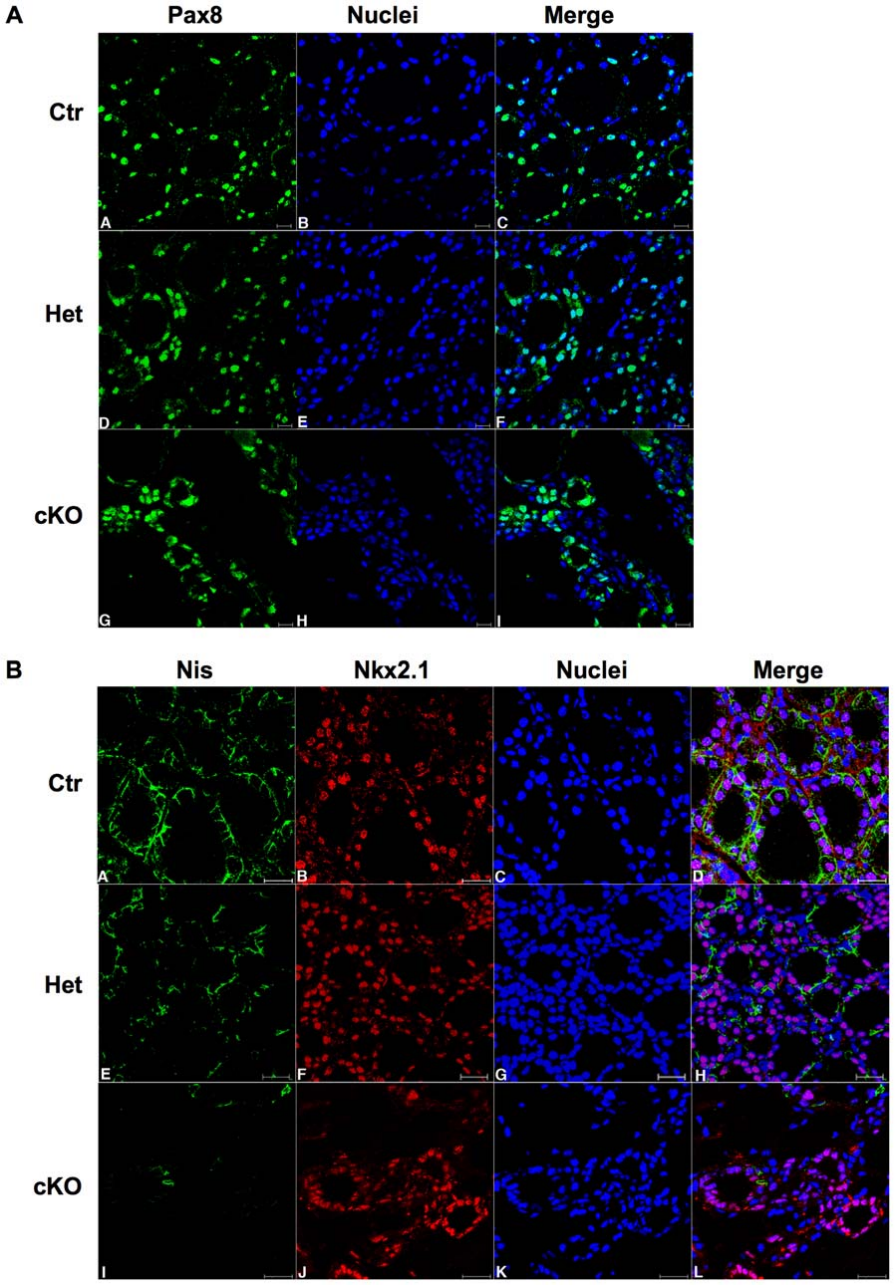
Immunofluorescence analysis of differentiation in Dicer cKO thyroids at one month after birth. (A) Immunofluorescence analysis of Pax8 (green signal) in Ctr (A–C), Het (D–F) and cKO (G–I) thyroids at 1 month (scale bar: 20 µm). Nuclei are stained with DRAQ5 (blue signal). (B) Double immunofluorescence analysis of Nis and Nkx2.1 (green and red signal, respectively) in Ctr (A–D), Het (E–H) and cKO (I–L) thyroids at 1 month (scale bar: 20 µm). Nuclei are stained with DRAQ5 (blue signal). Ctr denotes Dcr1^Flox/Flox^ mice, used as controls.

**Figure 6:**
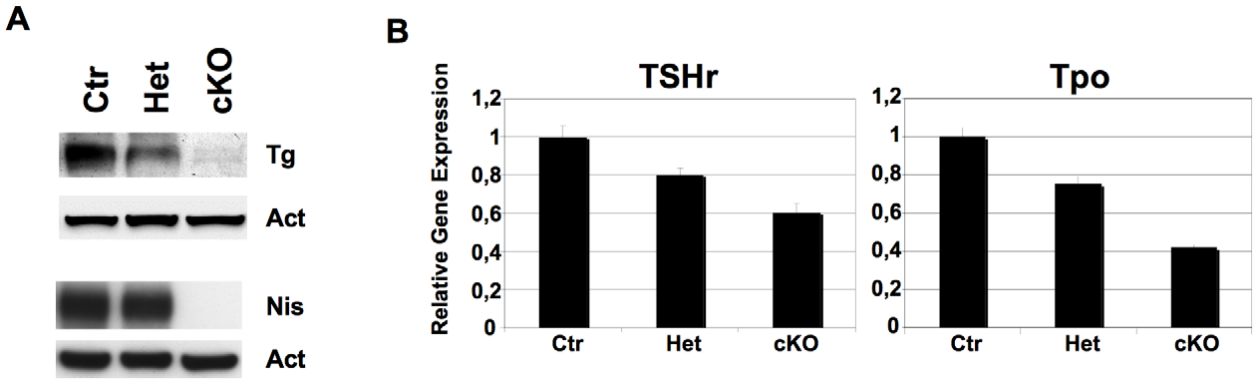
Late differentiation markers expression in Dicer cKO thyroids at one month after birth. (A) Western blot analysis of Tg and Nis proteins in Ctr, Het and cKO thyroids. (B) Q-PCR analysis of TSHr and Tpo expression in Ctr, Het and cKO thyroids at 1 month. Each RNA sample is obtained by pooling four thyroids/genotype. Ctr denotes Pax8^Cre/+^ mice, used as controls.

The expression of several thyroid differentiation markers was analyzed by immunofluorescent staining, showing that the two transcription factors Pax8 and Nkx2.1 were still present, while the Na/I symporter Nis was absent (fig. 5A and B). Western blot analysis confirmed the loss of Nis expression and revealed a strong repression in the expression of Tg in one month mutant mice (fig. 6A), in sharp contrast with the normal Tg expression observed in newborn mice (fig. 3B).

We also measured by Q-PCR the expression of two additional proteins essential for proper thyroid gland function, TSH receptor (TSHr) and Tpo. Figure 6B shows that the transcripts were reduced by 40% for TSHr and 60% for Tpo in cKO mice (fig. 6B). Interestingly both mRNAs were also reduced, albeit at lesser extent, in heterozygous mice (fig. 6B). Taken together, these data demonstrate that in the adult thyroid Dicer contributes to the maintenance of thyrocyte differentiation by regulating the expression of several key genes.

**Figure 7:**
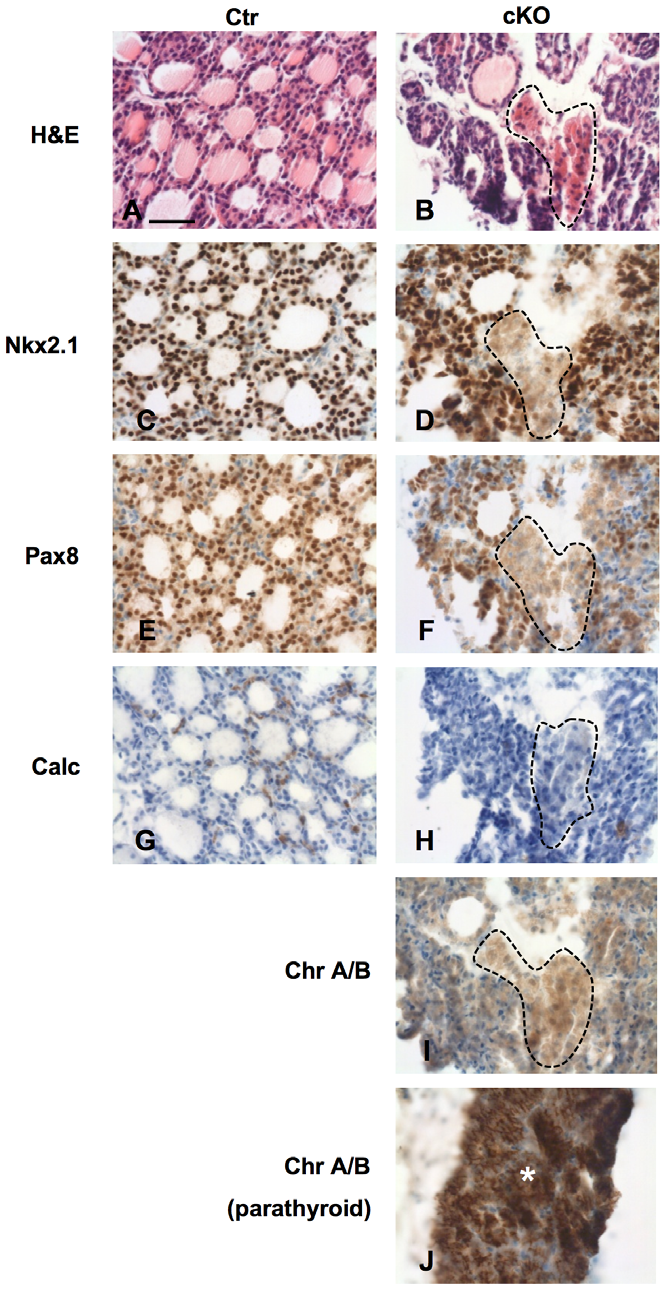
Oxyphilic cells in postnatal Dicer cKO thyroid gland do not express thyroid markers. Hematoxylin and eosin (H&E) staining (A, B) and immunohistochemical analysis of Nkx2.1 (C, D), Pax8 (E, F), Calcitonin (Calc) (G, H) in Ctr and cKO thyroid sections at 1 month. Immunohistochemical analysis of Chromogranin A/B expression is shown in thyroid (I) and in chief cells of the parathyroid glands (J, indicated with *) of cKO mice (scale bar: 50 µm). Dashed lines delimit representative oxyphilic cells patches.

The oxyphilic cells noticed in Dicer cKO thyroids remnants did not show any immunoreactivity for Nkx2-1 or Pax8, in contrast to the remaining cells with follicular differentiation. They are furthermore negative for markers of thyroid C-cells (calcitonin) and of cells of the parathyroid glands (chromogranin) (fig. 7).

### The expression of cell adhesion proteins is dysregulated by Dicer inactivation

Thyroid functionality is strictly dependent on cell polarity, with segregation of proteins between the apical and the basolateral membrane compartments. In order to assess if Dicer inactivation influences cell polarity, we analysed the localization and the amount of two cell-cell adhesion proteins belonging to the cadherin family, Cdh16 (ksp-cadherin) and Cdh1 (E-cadherin), both of them being normally expressed in the basolateral membrane.

**Figure 8:**
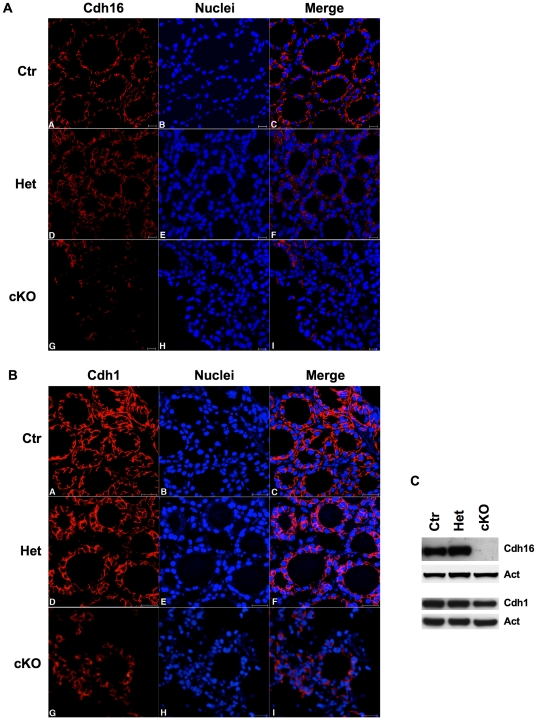
Dicer inactivation affects expression of thyrocyte cell adhesion proteins. (A) Immunofluorescence analysis of Cdh16 (red signal) in Ctr (A–C), Het (D–F) and cKO (G–I) thyroids at 1 month (scale bar: 20 µm). Nuclei are stained with DRAQ5 (blue signal). (B) Immunofluoresence analysis of Cdh1 (red signal) in Ctr (A–C), Het (D–F) and cKO (G–I) thyroids at 1 month (scale bar: 20 µm). Nuclei are stained with DRAQ5 (blue signal). (C) Western blot analysis of Cdh16 and Cdh1 proteins from Ctr, Het and cKO thyroids at 1 month. Ctr denotes Dcr1^Flox/Flox^ or Pax8^Cre/+^ mice, used as controls.

Immunofluorescence analysis evidenced a strong repression of Cdh16 expression (fig. 8A), while Cdh1 is still expressed, albeit with a reduced membrane localization (fig. 8B). Western blot analysis confirmed the loss of Cdh16 and the mild downregulation of Cdh1 levels (fig. 8C). These data demonstrate that in Dicer mutant mice the expression of markers of thyroid polarity are either reduced or lost.

## Discussion

In this paper we demonstrate that the micro-RNA processing enzyme Dicer is essential for several aspects of postnatal thyroid gland function and structure. Mice with thyroid-specific ablation of Dicer develop a pronounced impairment of thyroid function leading to shortened life span. The observed high serum TSH level and reduced body weight are indicative of severe hypothyroidism in the knockout mice.

Interestingly, no gross alterations of thyroid gland localization, morphology and differentiation are observed during embryonic development and in newborn mice. Dicer conditional knockout mice have been obtained using Pax8^Cre/+^ mouse line [25], in which Cre recombinase is controlled by the Pax8 locus, that is active in the in thyroid from E8.5 and throughout the adult life. Thus, Dicer-dependent miRNAs are supposed to be ablated from E8.5 and consequently also during migration of the thyroid bud from the pharyngeal floor to its final position in front of the trachea, as well as during the completion of thyrocyte differentiation. Although we cannot exclude that the rate of Cre-mediated Dicer inactivation at early developmental stages might not be sufficient to completely abolish the expression of miRNAs, these data argue for the possibility that miRNAs are dispensable during initial stages of thyroid morphogenesis.

Molecular analysis of knockout thyroid glands at 1 month after birth shows a strongly reduced expression of late differentiation markers such as Nis and Tg, while the expression of the early differentiation markers Pax8 and Nkx2.1 appears unaffected. Furthermore the epithelial polarity markers Cdh1 and Cdh16 are downregulated, thus correlating with the loss of polarized follicular architecture. As it has been previously demonstrated that thyroid-specific Cdh1 knockout mice still retain thyrocyte polarization [24], the reduction of Cdh1 membrane localization might be a consequence, rather than causative, of the compromised cell polarity. Hypothetically also loss of Nis protein expression could be a consequence of altered thyrocyte polarity.

It is well known that Pax8 and Nkx2.1 transcription factors positively regulate Tg transcription in vitro, and that both are necessary for Tg expression *in vivo* [26], [27]. The existence of a developmental stage between E8.5 and E15.5 in which Tg is still absent despite the presence of both transcription factors, however, demonstrates that Pax8 and Nkx2.1 are not sufficient to switch-on the expression of Tg. In Dicer cKO mice Tg and Nis are strongly reduced after birth while the early differentiation markers Pax8 and Nkx2.1 show a normal pattern of expression. This observation suggests that Dicer could control unknown events regulating the transition between the early and the late differentiation of thyroid gland. We can speculate that the absence of an intact miRNAs processing machinery drives the thyroid epithelial cells towards a less differentiated status in which the expression of transcription factors is uncoupled from the expression of their well-established target genes.

In accordance with what has been reported for other tissues where Dicer has been conditionally inactivated [9], [12], [28], thyroid size is clearly reduced when Dicer is ablated. Importantly, the reduced organ size is not correlated with increased apoptosis and could be due, at least in part, to the reduction of thyroglobulin, the main component of the colloid, in mutant thyroids. Moreover, in homozygous mice, a population of oxyphilic cells lacking either thyroid or parathyroid differentiation markers appears. Reduced Dicer expression in breast cancer make epithelial cells to adopt a less-differentiated fate [29]. Also thyroid neoplastic transformation is associated with reduced miRNAs expression and loss of differentiation [30]. This makes it attractive to hypothesize that the oxyphilic cells are likely to constitute a (pre-) neoplastic population of thyrocytes lacking differentiation features.

Taken together, our results demonstrate for the first time that the microRNA-processing enzyme Dicer is essential for thyroid function, and pave the way to the identification of specific microRNAs playing key roles in thyroid physiology.

## Materials and Methods

### Ethics statement

All the procedures involving animals were conducted as indicated in the Italian National Guidelines (D.L. No. 116 G.U., suppl. 40, 18.2.1992, circolare No. 8, G.U. July 1994) and in the appropriate European Directives (EEC Council Directive 86/609, 1.12.1987), adhering to the Guide for the Care and Use of Laboratory Animals (United States National Research Council, 1996). All the *in vivo* experimental activities were approved by the Animal Ethics Committee (CESA) of Biogem (Italy) with ID no. 0908.

### Generation of Dcr1^Flox/Flox^;Pax8^Cre/+^ mice

Mice with a floxed Dicer (Dcr) allele, Dcr1^Flox/Flox^ mice [31], were crossed with Pax8^Cre/+^ mice [30]. Double heterozygous offspring (Drc1^Flox/+^;Pax8^Cre/+^) were mated with Drc1^Flox/Flox^ mice to obtain Drc1^Flox/Flox^;Pax8^Cre/+^. Mice used in this study were housed and maintained in pathogen free conditions. All animal experiments were performed according to approved institutional protocols.

### Genotyping

Genomic DNA was isolated from tail snips, yolk sacs or thyroids in lysis buffer (50 mM Tris HCl, 100 mM EDTA, 100 mM NaCl, 1% SDS) and 0,5 mg/ml proteinase K. PCR genotyping was performed with three different primers to discriminate between Pax8^Cre^ and Pax8^+^ alleles [25] and the following primers diagnostic for Dcr1^Flox^ and wild-type Dcr1 alleles: Dcr1 *forward* 5′-ATTGTTACCAGCGCTTAGAATTCC-3′ and R2 5′-GTACGTCTACAATTGTCTATG-3′. Cre site specific recombination of Dicer floxed allele was confirmed by PCR using the primers R2 and F2 5′-TCGGAATAGGAACTTCGTTTAAAC-3′, while Dicer exon 21 was amplified using the primers F1 5′-ATACCCTAACTTAGACTTCG-3′ and R1 5′-AAGAGTCCTTGAGGAGTACC-3′.

### Histology and immunohistochemistry

Animals were killed by CO_2_ asphyxia. Thyroids and embryos were fixed overnight (o. n.) at 4°C in 4% paraformaldehyde in PBS, pH 7.2, dehydrated through ethanol series, cleared in xylene and embedded in paraffin. For histological analysis, 7 µm sections were stained with hematoxylin and eosin (Sigma-Aldrich, St. Louis, MO), according to the manufacturer's instructions.

For immunohistochemical analysis, 7 µm sections were dewaxed by standard techniques. Heat treatment was performed for antigen retrieval in sodium citrate buffer, pH6, followed by permeabilization by incubating sections in PBS containing 0,2% Triton X-100. Incubation with primary antibodies was performed o.n. at 4°C in blocking buffer (3% BSA, 5% goat serum, 20 mM MgCl_2_, 0,3% Tween 20 in PBS). Endogenous peroxidase activity was quenched with 35% H_2_O_2_ in methanol at room temperature and chromogenic reactions were carried out according to the Vectastain ABC kit protocol (Vector Laboratories, Burlingame, CA) with or without haematoxylin as nuclear counterstain.

The following primary antibodies were used at the indicated concentrations/dilutions: anti-Pax8 rabbit polyclonal antibody 0,87 µg/ml [26]; anti-Nkx2.1 rabbit polyclonal antibody 0,6 µg/ml [32]; anti-Calcitonin rabbit polyclonal antibody 1∶2000 (Dako, Milan, Italy), anti-chromogranin A/B rabbit polyclonal antibody (Eurodiagnostica, Malmö, Sweden). Biotinylated anti-rabbit IgG 1∶200 was used for detection of primary antibodies (Vector Laboratories, Burlingame, CA).

### Immunofluorescence

Deparaffinization, hydration and antigen retrieval of tissue 4 µm sections were performed by means of wax capture (W-CAP) antigen retrieval solution, pH 6.0, according to manufacturer's instructions (Bio-Optica, Milan, Italy). Tissue sections were incubated o. n. at 4°C with primary antibody diluted in blocking buffer. Nuclei were stained with the DNA intercalator DRAQ5 (Alexis Corporation, Lausen, Switzerland).

Immunofluorescence analysis was performed at a confocal laser scanning microscope LSM 510 Meta (Zeiss, Gottingen, Germany). The lambda of the argon ion laser was set at 488 nm, that of the two HeNe lasers was set at 546 and at 633 nm. Fluorescence emission was revealed by BP 505–530 band pass filter for Alexa Fluor 488, by BP 560–615 band pass filter for Alexa Fluor 546 and by 615 long pass filter for DRAQ5. Double staining immunofluorescence images were acquired separately in the green, red and infrared channels at a resolution of 1024×1024 pixels, with the confocal pinhole set to one Airy unit, and then saved in TIFF format.was performed at a confocal laser scanner microscope (LSM 510; Zeiss, Göttingen, Germany). The lambda of the argon ion laser was set at 488 nm, and that of the HeNe laser was set at 543 nm. Fluorescence emission was revealed by BP 505–530 band pass filterfor Alexa Fluor 488 and by BP 560–615 band pass filter for Alexa Fluor 546.

The following primary antibodies were used at the indicated concentrations/dilutions: anti-Nkx2.1 mouse monoclonal antibody 1∶100 (Thermo Fisher Scientific, Fremont CA-USA); anti-Nis rabbit polyclonal antibody 0.096 µg/ml; anti-Ksp-Cadherin rabbit polyclonal antibody 1∶100 (Zymed Lab Inc) and anti-Cdh1 1∶100 mouse monoclonal antibody (BD Transduction Laboratories, Lexington, KY, USA).

Biotinylated anti-rabbit IgG 1∶200, Fluorescein Anti-Rabbit IgG and Rhodamin Anti-mouse IgG were used for detection of primary antibodies (Vector Laboratories, Burlingame, CA).

### TSH measurements

Venous blood samples were collected in microtubes without anticoagulant. After clot formation, the samples were centrifuged, and the serum fraction was kept at −20°C. TSH levels were determined by an ELISA kit (Gentaur, Brussels, Belgium).

### Western blot

Pools of four thyroids/genotype were homogenized with a potter and lysed in 50 mM Tris, 150 mM NaCl, 0,1% SDS, 1% Triton X-100, 5 mM MgCl_2_, 0,5% deoxicholic acid, 0,5 mM Na_2_P_2_O_7_, 0,5 mM PMSF, 1 mM DTT, 50 mM NaF, 0,5 mM Na_3_VO_4_ with the addition of protease inhibitors. Protein concentration was measured by the BCA protein assay reagent (Pierce, Rockford, IL) according to manufacturer's instructions.

Western blots were performed as previously described [33]. The following primary antibodies were used at the indicated concentrations/dilutions: anti-β-actin mouse monoclonal antybody 1∶5000 (Sigma-Aldrich, St. Louis, MO); anti-Tg rabbit polyclonal antibody 1∶10000 (Dako, Milan, Italy); anti-Nis rabbit polyclonal antibody 0,24 µg/ml; anti-Ksp-Cadherin mouse monoclonal antibody 1∶1000 (USBiological) and anti-Cdh1 1∶1000 mouse monoclonal antibody (BD Transduction Laboratories Lexington, KY, USA). Immune complexes were detected by enhanced chemiluminescence (ECL) as instructed by manufacturer (Amersham Biosciences, Piscataway, NJ). Chemiluminescence was captured and analyzed with a Chemidoc Xrs supported by the Quantity One 4.6.1 software (Bio-Rad Laboratories, Hercules, CA).

### Quantitative PCR

Total RNA was extracted from pools of four thyroids/genotype using TRIzol Reagent (Invitrogen Life Technologies, Carlsbad, CA) according to manufacturer's instructions. Q-PCR analysis was performed using 7900 Fast Real Time PCR System (Applied Biosystems, Foster City, CA).

Quantitative analysis of miRNA expression was performed by RNA retrotranscription and subsequent TaqMan real-time PCR, as recommended by the supplier (Applied Biosystems, Foster City; CA). For each retrotranscription reaction 10 ng of total RNA were used. Results were expressed as a cycle threshold (CT) value. Normalized CT values were obtained by subtracting the CT value (averaged across two replicates) of the control nucleolar RNA snoRNA202 from the raw CT value of miRNAs.

Quantitative analysis of TSHr and Tpo expression was perfomed on total cDNA obtained with the Superscript II Reverse Transcriptase kit (Invitrogen Life Technologies, Carlsbad, CA) according to the instructions of the manufacturer and using random hexamers a primers. The PCR reaction mixture contained 10 ng of total cDNA, Power PCR Master Mix 1× (Applied Biosystems, Foster City; CA) and the following primers used at a concentration of 300 nm each: ; TSHrFw: 5′-TCCCTGAAAACGCATTCCA-3′ and TSHrRev: 5′-GCATCCAGCTTTGTTCCATTG-3; ’TpoFw: 5′-CAAAGGCTGGAACCCTAATTTCT-3′ and TpoRev: 5′-AACTTGAATGAGGTGCCTTGTCA-3′; RNA18SFw:5′-CGGCTACCACATCCAAGGAA-3′ and RNA18SRev:5′-GGGCCTCGAAAGAGTCCTGT-3′.

All the reactions were carried out in triplicate. For each sample, the expression of the genes of interest was normalized for the expression of RNA18S gene measured under the same condition and corrected for primers amplification efficiency.
